# RETRACTION: TLR4 Modulates Senescence and Paracrine Action in Placental Mesenchymal Stem Cells via Inhibiting Hedgehog Signaling Pathway in Preeclampsia

**DOI:** 10.1155/omcl/9805286

**Published:** 2025-09-11

**Authors:** Oxidative Medicine and Cellular Longevity

RETRACTION: Y. Zhong, Y. Zhang, W. Liu, Y. Zhao, L. Zou, and X. Liu, “TLR4 Modulates Senescence and Paracrine Action in Placental Mesenchymal Stem Cells via Inhibiting Hedgehog Signaling Pathway in Preeclampsia,” *Oxidative Medicine and Cellular Longevity* 2022 (2022): 7202837, https://doi.org/10.1155/2022/7202837.

The above article, published online on 14th June 2022 in Wiley Online Library (wileyonlinelibrary.com), has been retracted by agreement between the authors; the journal Editor-in-Chief, Jeannette Vasquez-Vivar; and John Wiley & Sons Ltd. The retraction has been agreed due to figure concerns initially raised by the authors. Specifically:

• The preeclampsia panel shown in Figure 1e is identical to the LPS panel shown in Figure 3g.
• There are unexpected, repeated elements between the NC group and the LPS+SAG^CM^ group in Figure 4c.

The results are therefore considered unreliable, and the article is retracted.

The authors agree with the retraction.

